# Predominant affective temperaments in depressive patients with severe social withdrawal

**DOI:** 10.1186/s12991-024-00496-z

**Published:** 2024-03-19

**Authors:** Laura Orsolini, Giulio Longo, Silvia Bellagamba, Takahiro A. Kato, Umberto Volpe

**Affiliations:** 1 Unit of Clinical Psychiatry, Department of Neurosciences/DIMSC, Polytechnic University of Marche, Via Conca 71, 60126 Ancona, Italy; 2 Department of Neuropsychiatry, Graduate School of Medical Sciences, Kyushu University, Fukuoka, Japan

**Keywords:** Affective temperament, Hikikomori, Social withdrawal, Temperaments, Youth, Youth mental health

## Abstract

**Background:**

Hikikomori (HK) is characterized by self-isolation and social refusal, being more likely also associated with affective disorders, including depression. This case–control study primarily aimed at identifying (if any) predominant affective temperaments are associated with HK in depressed versus not-depressed individuals. Secondary objectives comprise assessing which other psychopathological dimensions (e.g., boredom, anxiety) are associated with the HK specifier in depressed individuals.

**Methods:**

From the larger SWATCH study, 687 Italian young people were screened for depression, as measured by 9 items-Patient Health Questionnaire (PHQ-9) and HK-like social withdrawal, through the Hikikomori Questionnaire-25 (HQ-25). All subjects were administered a brief-Temperament Evaluation of Memphis, Pisa, Paris and San Diego (TEMPS-M), the 7 items-Generalized Anxiety Disorder (GAD-7) and the Multidimensional State Boredom Scale (MSBS).

**Results:**

Males reported significantly higher scores at HQ-25 total score than females (p = 0.026). In the total sample, HK social withdrawal is positively predicted by MSBS low arousal, disengagement, depressive levels, depressive and irritable affective temperaments, while negatively by anxiety (F(6, 680) = 82.336, p < 0.001, R^2^ = 0.421). By selecting only depressed sample, HQ-25 is positively predicted by MSBS total score, low arousal and depressive affective temperament, while negatively by MSBS high arousal (F(4, 383) = 48.544, p < 0.001, R^2^ = 0.336). The logistic regression model found that the likelihood of developing depression with the HK specifier is significantly predicted by depressive and cyclothymic affective temperaments.

**Conclusions:**

These preliminary findings could help in clinically characterizing the relationship between specific affective temperamental profiles among individuals with depression with/without HK specifier, in order to provide a more tailored and personalized therapeutic approach. Our Italian study should be extensively replicated in larger, longitudinal and multicentric pan-European studies, by specifically assessing the impact of these findings on depression clinical course, prognosis and treatment outcomes.

## Background

**Fig. 1:**
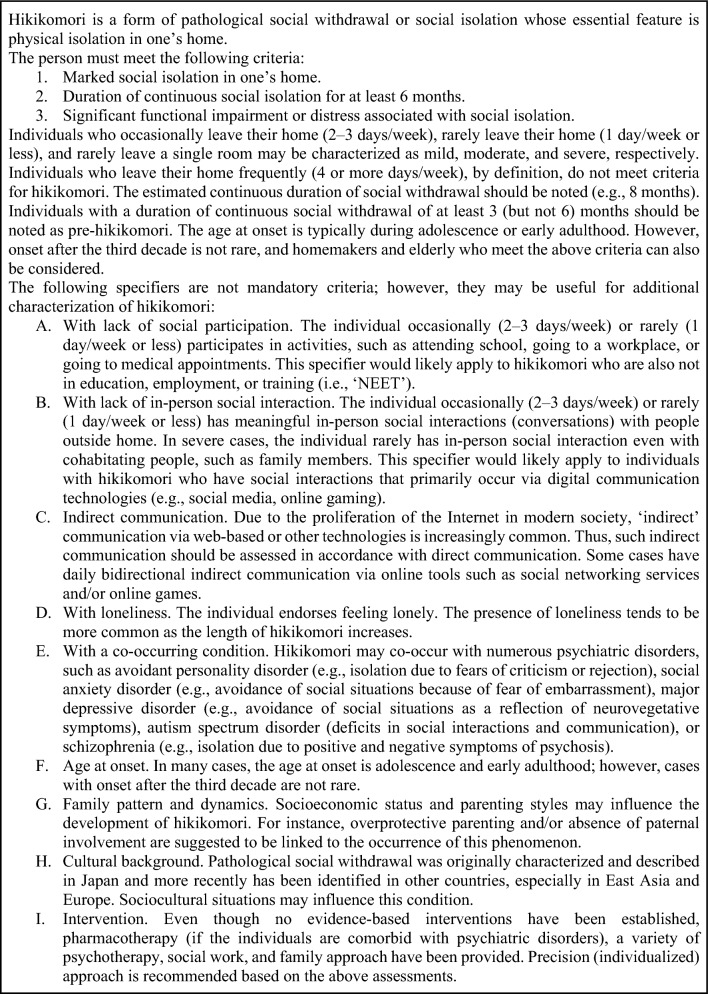
Diagnostic criteria for Hikikomori Syndrome (from Kato et al. [4]

During the last years, there has been an increasing interest in investigating and clinically characterizing certain newly described psychopathological entities in Western Countries, originally described only in oriental ones, such as in Japan [1, 2]. The Hikikomori syndrome (引き籠もり) was described for the first time in Japan and it has been considered for long time as a culture/Japan-bound syndrome [3, 4], even though nowadays evidence confirmed its spread as diagnostic entity in Western countries, including Italy [5–8]. Hikikomori, firstly described by the psychiatrist T. Saito in 1998 [9], refers to any individual who intentionally withdraws him/herself into his/her home or room for at least 6 months, refusing any social situation and interaction with all people in-person, including friends and/or relatives [4]. Etymologically, it derives from the Japanese composed verb, formed by “*hiku*” (i.e., “*to pull back*”) and “*komoru*” (i.e., “*seclude oneself*”) [4]. Hikikomori syndrome could be classified as a primary (or idiopathic) or secondary. The primary form is not associated with other diseases. While secondary form is associated with other (psychiatric or not) illnesses [4]. Diagnostic criteria for Hikikomori are described in Fig. 1.

Indeed, social withdrawal has been identified to be associated with several psychiatric conditions such as psychotic disorders, personality disorders and affective disorders (mainly major depression) [10, 11]. Furthermore, despite Hikikomori has been described in Japan for more than 20 years, there are few studies specifically investigating psychopathological, personality and temperamental characteristics of individuals with Hikikomori-like social withdrawal [2, 12–15]. Pioneering studies are focusing on various psychological factors frequently reported among hikikomori subjects such as the lack of functional coping strategies, interpersonal difficulties, low self-esteem levels, poor autonomy capacity, tendency to experience anxious-depressive states, environmental sensitivity, and insecure/anxious attachment pattern [13, 16–18]. Indeed, determining which are the typical/predominant/predisposing hikikomori temperamental patterns, also considering sex-based differences, would be important not only to allow an early and precise clinical characterization of the phenomenon also in Italy, but also to predict potentially risky psychopathological trajectories such as suicidal behaviours as well as personalizing more tailored interventions [1, 15, 19]. However, currently there are no studies specifically conducted to explore the role of predominant affective temperaments in the onset and maintenance of Hikikomori-like social withdrawal symptomatology.

The concept of affective temperament refers to the emotional domain of personality, which is related to the subject's predisposition to the development of mood disorders [20] and, potentially, also to those depressive pictures associated with Hikikomori-like social withdrawal symptomatology. Temperament is defined as a stable personality trait and refers to the activity levels, social and biological rhythms, mood and daily variability of patients [21]. According to Akiskal, all mental disorders, including both affective and psychotic conditions, have been supposed to represent abnormal forms of normal temperamental traits [22]. Indeed, Kraepelin was the first to identify affective temperaments, recognising the depressive, irritable, manic, and cyclothymic temperamental dispositions [22]. While Kretschmer identified the cyclothymic temperament (which combines all those affective temperaments previously theorized by Kraepelin) and its opposite schizotymic affective temperament [22]. Akiskal was indeed the first to clearly characterize and define five affective temperaments (i.e., cyclothymic, depressive, hyperthymic, irritable, and anxious) and to investigate the associations between specific predominant affective temperaments with the variable clinical characterization of mood disorders [21].

Overall, due to the extremely need to clinically characterize Hikikomori individuals, and, particularly, in stratifying depressed individuals with or without Hikikomori as diagnostic specifier, by investigating the potential association with specific predominant affective temperaments, according to the Akiskal’s classification, we carried out a sub-analysis within the larger SWATCH (Social Withdrawal And TeCno-mediated mental Health issues) study. Specifically, our main hypothesis to be tested consisted in assessing whether it is possible to identify (if any) specific predominant affective temperaments are more likely associated with the presence versus absence of the Hikikomori specifier to depression in order to provide a diagnostic subtyping of young depressed individuals useful for considerations regarding a more tailored and personalized therapeutic approach. Therefore, the primary objective of the present study aimed at investigating which predominant affective temperamental profiles are prevalent within a sample of Italian young people stratified according to the presence/absence of clinically significant depressive symptomatology with or without the Hikikomori diagnostic specifier. Secondary objectives comprise assessing whether there sex-based differences and whether other variables could be variably associated with the presence of the Hikikomori specifier among depressed individuals and a set of supposed more predominant associated affective temperaments, such as the boredom dimension and the associated anxiety symptomatology.

## Methods

### Study design and recruitment strategies

The study was carried out by recruiting a sub-sample of Italian young people (aged 18–35) during the timeframe March-October, 2022, within the larger SWATCH study aimed at investigating the main psychopathological determinants of the severe youth social withdrawal condition (hikikomori-like) and web-based psychopathologies in Italian adolescents/young adults. From the SWATCH dataset, the following inclusion criteria were considered: (a) age ranging from 18 to 35 years-old; (b) written informed consent to participate in the current study. All participants who did not agree to provide a written informed consent and those who did not fully complete all questionnaires were properly excluded by the analysis. The final sample was stratified in two groups: subjects with clinically relevant depressive symptomatology (DEP +) and subjects without clinically relevant depressive symptomatology (DEP-), by using the 9-item Patient Health Questionnaire (PHQ-9) cut-off of 10 or above [23]. Both groups were homogeneous according to the sex and age. Sample size was calculated using the Statistical Software G*Power version 3.1. (Franz, Universitat Kiel, Germany), by keeping the values of confidence level as 99%, anticipated population proportion 0.5, an α error of 0.05, a power of 80%, and taking into consideration all variables to be entered in the multivariable analysis, in order to obtain at least an effect size of > 0.3. A minimum total sample size of 278 was established to be reached for the present study, divided in two groups (DEP + and DEP-) constituted by at least 139 participants each one. The study was conducted in accordance with the ethical principles outlined in the Declaration of Helsinki and according to the guidelines for Good Clinical Practice (GCP) (WHO, 2013), following the approval by the local Institutional Review Board. All participants gave informed consent to take part in the study.

### Measurements

A case report form (CRF) was developed to collect a set of socio-demographic and clinical characteristics, including participants' age, sex, occupational and marital status, living condition, parents’ marital status, and previous psychiatric history. All participants were administered at baseline the following self-administered questionnaires, as described below.

The PHQ-9 is a 9-items tool designed to screen for depression in primary care and specialty medical settings [23]. The standard cutoff score to identify possible major depression is 10 or above, with a sensitivity of 88% and a specificity of 88% for major depression [23]. The PHQ-9 total score obtained by summing all 9 items, allows clinicians to discriminate between mild (PHQ-9 ranging 10–14), moderate (PHQ-9 ranging 15–19) and severe (PHQ-9 ≥ 20) depression [24]. The instrument has a good reliability in identifying clinically relevant depressive symptomatology also in the Italian sample [25]. In our study, Cronbach's α of the PHQ-9 showed a satisfactory internal reliability (α = 0.849).

The Generalized Anxiety Disorder-7 (GAD-7) is a 7-items questionnaire primarily identified for screening of GAD suitable to individuation of anxious patterns of symptoms [26]. The standard score to identify clinically significant anxiety indicative of a generalized anxiety disorder is 10 or above, with a sensitivity of 89% and a specificity of 82%, while the cut-off of 5 or above was established to identify significant clinically relevant anxiety symptomatology [26]. The GAD-7 total score obtained by summing all 7 items, allows clinicians to discriminate between mild (GAD-7 ranging 5–9), moderate (GAD-7 ranging 10–14) and severe (GAD-7 ≥ 15) anxiety [26]. In our study, Cronbach’s α of the GAD-7 showed satisfactory internal reliability (α = 0.887).

The 25-item Hikikomori Questionnaire (HQ-25) is a 25-item self-report scale measuring symptoms of Hikikomori-like social withdrawal over the past 6 months. For each item, response options range from 0 “strongly disagree” to 4 “strongly agree” [27]. The HQ-25 provides a three-factor theoretical model of Hikikomori construct, namely socialization, isolation and emotional support. A total HQ-25 score was obtained by summing up individual items’ scores. In the original and Italian study, the standard cutoff score to discriminate between individuals at risk for Hikikomori and those not at risk is 42 or above, with a sensitivity of 94% and a specificity of 61% [27, 28]. HQ-25 showed a good internal consistency, with Cronbach’s α values ranging 0.88 to 0.96 for the total scale, while 0.94, 0.91 and 0.88, respectively, for the three abovementioned subscales [27]. Reliability and validity of this instrument has been tested also in Italian samples and reported good results, by confirming that the originally proposed Japanese three-factor measurement model structure of the HQ-25 and total score could be adapted in the Italian context as well [28]. In our study, Cronbach's α of the HQ-25 showed a satisfactory internal reliability (α = 0.813).

The brief version of the Munster Temperament Evaluation of Memphis, Pisa, Paris and San Diego (TEMPS-M) [29] is a 35-items self-report questionnaire used to assess affective temperaments described by Akiskal [30, 31], i.e. depressive, anxious, hyperthymic, cyclothymic and irritable, using a dimensional approach with a 5-point Likert scale ranging from 1 (“not at all”) to 5 (“very much”) [29]. TEMPS-M has been developed in different clinical and research settings [32]. In our study, Cronbach's α of the TEMPS-M total score and depressive, cyclothymic, hyperthymic, irritable and anxious showed satisfactory internal reliability (respectively, α = 0.898, α = 0.884, α = 0.903, α = 0.841, α = 0.853, α = 0.803).

The Multidimensional State Boredom Scale (MSBS) consists of 29 items with answers on a seven-point Likert scale, from 1 (“strongly disagree”) to 7 (“strongly agree”) [33]. The items are divided into five factors/subscales: (a) time perception (TP), which describes the slow passage of time; (b) disengagement (DIS), regarding a lack of involvement; (c) inattention (INA), or difficulty focusing attention on events; (d) high arousal (HA), which concerns the negative effects of an excessively high arousal; and (e) low arousal (LA), which covers the experiences and behavior attributable to an excessively low arousal. Scores obtained for these five factors are combined to obtain an overall boredom score. The instrument has been also validated in Italian samples [33], by showing good psychometric properties, including an excellent internal consistency. In our study, Cronbach's α of the MSBS also showed an excellent internal reliability (α = 0.965).

### Statistical analyses

All analyses were performed using the software Statistical Package for Social Science (SPSS) per MacOS version 28.0 (IBM SPSS Statistics, Chicago, IL, United States). For all analyses, the level of statistical significance was set at *p* < 0.05, two-tailed. Descriptive statistics were performed in order to describe the socio-demographic and clinical characteristics of the sample. Categorical variables are summarized as frequency (n) and percentage (%) whilst continuous variables as means [standard deviation (SD)]. The normality of the distribution of all continuous variables were verified by using skewness, kurtosis and the Kolmogorov–Smirnov test, and the equality of variances by Levene test. The total sample was initially divided into two groups: DEP + and DEP-. Then, the sample was also stratified into four groups, depending on the presence or absence of clinically significant depression (as measured through PHQ) and the presence or absence of specifier Hikikomori (as measured through HQ-25): group 1 (DEP + /HK +), group 2 (DEP + /HK-), group 3 (DEP-/HK +) and group 4 (DEP-/HK-). To compare all socio-demographic and categorical variables in each group, the *χ*^2^ Test was used. While Student’s t-test and two-way tailored analysis of variance (ANOVA) were performed, respectively, to compare all continuous variables between DEP + versus DEP- groups and between four groups (DEP + /HK + ; DEP + /HK-; DEP-/HK + ; DEP-/HK-), after verifying the normality of quantitative variables. Primary outcome was evaluating which predominant Akiskal’s affective temperament is more likely associated with Hikikomori-like social withdrawal specifier (as measured by HQ-25) in the total sample and within the depressive versus not-depressed subsample. Bivariate Person’s correlations have been used to investigate potential associations between the primary outcome and other variables, particularly TEMPS-M, MSBS, GAD-7 and PHQ-9. Multivariate linear regression models have been assessed to investigate variables associated with the severity of Hikikomori-like social withdrawal symptomatology, including as independent variables depressive symptomatology (as measured by PHQ-9), anxiety symptomatology (as measured by GAD-7), Akiskal’s affective temperaments (as measured by TEMPS-M), boredom dimension (as measured by MSBS and its subscales). Multivariate linear regression models have been performed in the total sample, with also a subanalysis across both sexes (males versus males), in order to investigating potentially sex-based differences in the findings, and within the depressed sample only, in order to investigate whether (if any) differences were found considering the presence of depressive symptomatology. Then, a binary logistic regression analysis was run within the sample of individuals with depression, to evaluate which predominant affective temperament are associated with a concomitant Hikikomori-like social withdrawal symptomatology. The odds ratios (OR), corresponding to 95% of confidence intervals (CI), standardized coefficient β values were generated for each variable.

## Results

### Socio-demographic characteristics of the sample

A sample of 687 outpatients young adults was included in the study (Table 1). Participants’ mean age was 24.1 (SD = 3.2), without sex-based differences (p = 0.671). Around two-third of the sample is represented by females (N = 523; 76.1%). Most participants declared to have a stable affective relationship (N = 637; 92.8%) and to live with his/her family members (N = 372; 54.1%). Most of the sample declared that their parents are not separated and/or divorced (N = 537; 78.2%). Most of the sample declared a previous positive psychiatric history (N = 524; 76.3%). The mean education level (in years) was 17.6 (SD = 2.5), without sex-based differences (p = 0.546).

**Table 1:** Socio-demographic characteristics of the sample

	Total sample	DepHK	DepHK-	noDepHK +	noDepHK-	p-value
Sex						
Males	164 (23.9%)	62 (25.4%)	26 (18.1%)	31 (34.8%)	45 (21.4%)	χ^2^ = 9.570 **p = 0.023**
Females	523 (76.1%)	182 (74.6%)	118 (22.6%)	58 (65.2%)	165 78.6%)	
Age (years)						
M (SD)	24.1 (3.2)	24.1 (3.2)	24.2 (3.4)	26.6 (3.1)	24.2 (3.2)	p = 0.625
Educational level (years)						
M (SD)	17.6 (2.5)	17.7 (2.4)	17.6 (2.7)	17.9 (2.6)	17.5 (2.2)	p = 0.521
Living condition						
With their nuclear family	372 (54.1%)	122 (50%)	72 (50%)	52 (58.4%)	126 (60%)	χ^2^ = 26.440 p = 0.190
With one their parents	63 (9.2%)	26 (10.7%)	17 (11.8%)	2 (2.2%)	18 (8.6%)	
With other relatives (not parents)	11 (1.6%)	4 (1.6%)	3 (2.1%)	1 (1.1%)	3 (1.4%)	
Alone	35 (5.1%)	13 (5.3%)	10 (6.9%)	6 (6.7%)	6 (2.9%)	
In a university hostel/boarding school	19 (2.8%)	9 (3.7%)	4 (2.8%)	5 (5.6%)	1 (0.5%)	
Together with friends	72 (10.5%)	31 (12.7%)	13 (9%)	9 (10.1%)	19 (9%)	
With their partner	76 (11.1%)	26 (10.7%)	20 (13.9%)	9 (10.1%)	21 (10%)	
Other	39 (5.7%)	13 (5.3%)	5 (3.5%)	5 (5.6%)	16 (7.6%)	
Psychological problem history						
None	163 (23.7%)					χ^2^ = 25.207 **p < 0.001**
Yes	524 (76.3%)					
Siblings						
Yes	568 (82.7%)	47 (19.3%)	25 (17.4%)	10 (11.2%)	37 (17.6%)	χ^2^ = 2.956 p = 0.401
No	119 (17.3%)	197 (80.7%)	119 (82.6%)	79 (88.8%)	173 (82.4%)	
Relationship status						
Single	12 (1.7%)	2 (1%)	2 (1.4%)	2 (2.3%)	6 (2.9%)	χ^2^ = 11.620 p = 0.236
In a stable relationship	370 (53.9%)	122 (50%)	80 (55.6%)	43 (48.3%)	125 (59.5%)	
In a unstable relationship	38 (5.5%)	14 (5.7%)	11 (7.6%)	4 (4.5%)	9 (4.3%)	
Married	267 (38.9%)	106 (43.3%)	51 (35.4%)	40 (44.9%)	70 (33.3%)	

In bold significant p-values

### Psychopathological characteristics of the sample

The mean score at PHQ-9 was 11.0 (SD = 5.8), without sex-based differences (p = 0.221). Regarding depressive symptomatology, the sample is slightly mainly represented by individuals with clinically relevant depressive symptomatology (N = 388; 56.5%), based on the stratification of the sample by using the PHQ-9 cutoff ≥ 10 (Table 2).

**Table 2:** Psychopathological features of the sample

Scale, M (SD)	Total sample	DepHK	DepHK-	noDepHK +	noDepHK-	p-value
PHQ-9 total score	11.0 (5.8)	16.1 (4.3)	13.4 (3.1)	6.4 (2.1)	5.5 (2.3)	F_(3,683)_ = 486.263 p < 0.001*
GAD-7 total score	10.3 (5.4)	13.9 (4.3)	12.2 (3.9)	5.9 (3.9)	7.2 (3.9)	F_(3,683)_ = 176.712 p < 0.001*
HQ-25 total score	42.1 (13.6)	55.1 (8.6)	32.7 (6.1)	49.9 (6.7)	30.2 (6.8)	F_(3,683)_ = 547.499 p < 0.001*
HQ-25 Socialization	18.5 (6.4)	24.4 (4.5)	14.4 (3.4)	21.4 (4.3)	13.1 (3.5)	F_(3,683)_ = 373.610 p < 0.001*
HQ-25 Isolation	11.8 (6.4)	17.4 (4.7)	7.6 (3.0)	15.6 (4.1)	6.4 (3.1)	F_(3,683)_ = 392.189 p < 0.001*
HQ-25 emotional support	11.9 (2.9)	13.3 (2.6)	10.7 (2.7)	12.9 (2.1)	10.7 (2.8)	F_(3,683)_ = 50.432 p < 0.001*
TEMPS-M depressive	20.9 (7.4)	25.7 (6.6)	21.8 (5.9)	19.2 (6.3)	15.6 (5.8)	F_(3,683)_ = 103.307 p < 0.001**
TEMPS-Mcyclothymic	20.1 (8.0)	25.0 (6.9)	22.0 (7.2)	17.4 (6.6)	14.1 (5.7)	F_(3,683)_ = 112.067 p < 0.001*
TEMPS-M hyperthymic	19.0 (6.0)	17.9 (6.2)	18.9 (5.4)	18.9 (6.0)	20.4 (6.0)	F_(3,683)_ = 6.832 p < 0.001**
TEMPS-M Irritable	14.9 (6.1)	17.3 (6.7)	15.2 (5.6)	14.1 (5.4)	12.5 (4.8)	F_(3,683)_ = 27.071 p < 0.001*
TEMPS-M anxious	19.5 (7.0)	22.7 (6.7)	20.5 (6.4)	18.0 (6.6)	15.7 (5.8)	F_(3,683)_ = 48.300 p < 0.001*
MSBS total score	104.2 (41.4)	136.8 (29.5)	102.0 (34.1)	96.1 (33.8)	71.7 (31.6)	F_(3,683)_ = 161.565 p < 0.001*
MSBS Disengagement	38.9 (16.4)	51.5 (11.6)	37.4 (13.9)	35.9 (13.9)	26.6 (13.1)	F_(3,683)_ = 143.205 p < 0.001*
MSBS high arousal	17.7 (8.1)	23.5 (6.7)	18.3 (7.0)	15.7 (6.3)	11.5 (5.9)	F_(3,683)_ = 132.424 p < 0.001*
MSBS inattention	17.6 (7.3)	22.0 (5.6)	17.9 (6.9)	16.2 (6.9)	12.7 (6.4)	F_(3,683)_ = 83.919 **p < 0.001**
MSBS low arousal	19.2 (9.3)	26.6 (6.9)	17.9 (7.6)	17.7 (8.0)	11.9 (6.6)	F_(3,683)_ = 166.017 **p < 0.001**
MSBS time perception	11.1 (7.0)	13.2 (8.4)	10.5 (6.3)	10.6 (5.8)	9.1 (5.5)	F_(3,683)_ = 14.586 **p < 0.001**

*HQ-25* hikikomori questionnaire-25 items, *PHQ-9* Patient health Questionnaire-9, *MSBS* multidimensional state boredom scale, *TEMPS-M* munster temperament evaluation of memphis, Pisa, Paris and San Diego; *GAD-7* generalized anxiety disorder-7

^*^Bonferroni

^**^T2 Tamhane

In bold significant p-values

The mean score at GAD-7 was 10.3 (SD = 5.4), with females who reported significantly higher anxiety scores, compared to males (p = 0.003). Regarding the anxiety symptomatology, the sample is highly represented by individuals who reported clinically relevant anxiety symptomatology (N = 569; 82.8%), based on stratification of the sample by using the GAD-7 cutoff ≥ 5 (Table 2).

The mean score at HQ-25 was 42.1 (SD = 13.6), with males who reported significantly higher scores at HQ-25 total score, compared to females (p = 0.026). The mean score at HQ-25 socialization subscale was 18.5 (SD = 6.4), without sex-based differences (p = 0.097). The mean score at HQ-25 isolation subscale was 11.8 (SD = 6.4), with slightly significantly higher scores among males (p = 0.042). The mean score at HQ-25 emotional support subscale was 11.9 (SD = 2.9), with significantly higher scores among males (p = 0.034) (Table 2).

The mean score at MSBS was 104.3 (SD = 41.4), without sex-based differences (p = 0.975). The mean score at MSBS Disengagement subscale was 38.9 (SD = 16.4), without sex-based differences (p = 0.290). The mean score at MSBS High Arousal subscale was 17.7 (SD = 8.1), without sex-based differences (p = 0.061). The mean score at MSBS Inattention subscale was 17.6 (SD = 7.3), without sex-based differences (p = 0.874). The mean score at MSBS Low Arousal subscale was 19.2 (SD = 9.3), without sex-based differences (p = 0.976). The mean score at MSBS Time Perception was 11.1 (SD = 7.0), without sex-based differences (p = 0.743) (Table 2).

The mean score at depressive temperament subscale at TEMPS-M was 20.9 (SD = 7.4), without significant sex-based differences (p = 0.191). The mean score at cyclothymic temperament subscale at TEMPS-M was 20.1 (SD = 8.0), without sex-based differences (p = 0.161). The mean score at hyperthymic temperament subscale at TEMPS-M was 19.0 (SD = 6.0), with males who reported significantly higher scores (p = 0.002). The mean score at irritable temperament subscale at TEMPS-M was 14.9 (SD = 6.1), without sex-based differences (p = 0.735) (Table 2). The mean score at anxious temperament subscale at TEMPS-M was 19.5 (SD = 7.0), with significantly higher scores among females (p ≤ 0.001).

### Variables associated with Hikikomori-like social withdrawal specifier within the depressed versus not-depressed sample

Considering all the total sample without distinguishing between depressive versus not-depressed individuals, according to the multivariate regression model, HQ-25 levels were positively predicted by MSBS low arousal levels (Beta coefficient, B = 0.428; 95%Confidence Interval, CI = (0.272)—(0.585); p < 0.001], MSBS disengagement (B = 0.145; 95%CI = (0.058)–(0.232); p = 0.001], depressive levels, as measured by PH9 total score (B = 0.482; 95%CI = (0.264)–(0.700); p < 0.001] and by irritable affective temperament (B = 0.215; 95%CI = (0.0075)–(0.325); p = 0.003] and depressive affective temperaments (B = 0.190; 95%CI = (0.043)–(0.338); p = 0.011]. While HQ-25 levels were negatively predicted by GAD-7 total score [B = − 0.252; 95%CI = (− 0.484)–(− 0.020); p = 0.033]. These variables statistically significantly predicted Hikikomori-like social withdrawal symptomatology (F(6, 680) = 82.336, p < 0.001, R^2^ = 0.421) (Table 3). Furthermore, we explored sex-based differences in Hikikomori-like social withdrawal symptomatology within the total sample, by splitting the multivariate regression linear model according to the sex (males versus females).

**Table 3:** Multiple linear regression with HQ-25 total score (as dependent variable)—total sample

	B	SE	β	t	p-value	95%IC lower limit	95%IC upper limit	Tolerance	VIF
MSBS Low Arousal	0.428	0.080	0.295	5.368	** < 0.001**	0.272	0.585	0.282	3.552
PHQ-9 total score	0.482	0.111	0.205	4.346	** < 0.001**	0.264	0.700	0.382	2.619
MSBS Disengagement	0.145	0.044	0.175	3.260	**0.001**	0.058	0.232	0.294	3.398
TEMPS-M Irritable	0.215	0.071	0.096	3.026	**0.003**	0.075	0.325	0.839	1.192
TEMPS-M Depressive	0.190	0.075	0.104	2.536	**0.011**	0.043	0.338	0.502	1.992
GAD-7 total score	− 0.252	0.118	− 0.100	− 2.137	**0.033**	− 0.484	− 0.020	0.387	2.581

*SE* standard error, *HQ-25* hikikomori questionnaire-25 items, *PHQ-9* patient health questionnaire-9, *MSBS* Multidimensional state boredom scale, *TEMPS-M* munster temperament evaluation of memphis, Pisa, Paris and San Diego; GAD-7: generalized anxiety disorder-7

In bold significant p-values

**Fig. 2:**
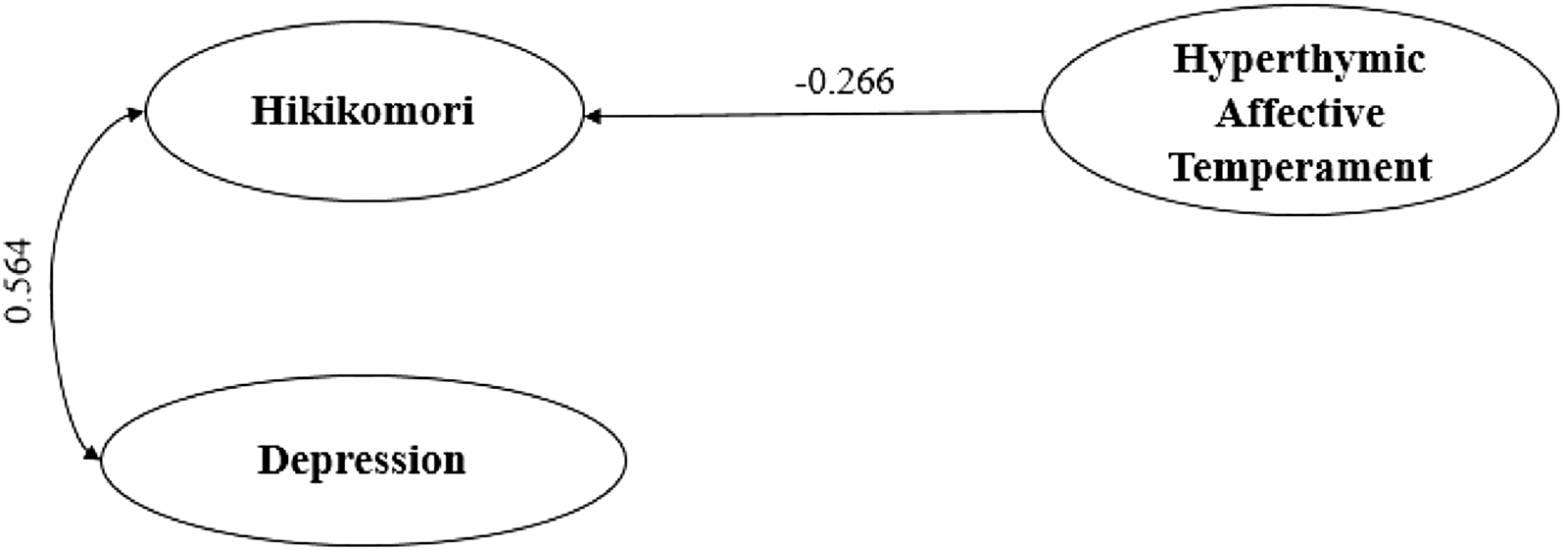
Graphical representation of relationships between Hikikomori, depression and affective temperaments (males)

According to the multivariate regression model, among males, HQ-25 levels were positively predicted by only MSBS total scores [B = 0.147; 95%CI = (0.092)–(0.202); p < 0.001] and depressive symptomatology, as measured by PHQ-9 [B = 0.564; 95%CI = (0.186)–(0.943); p = 0.004]. While, HQ-25 levels were negatively predicted by hyperthymic affective temperament [B = -0.266; 95%CI = (− 0.529)–(− 0.004); p = 0.047]. These variables statistically significantly predicted Hikikomori-like social withdrawal symptomatology (F(3, 160) = 37.520, p < 0.001, R^2^ = 0.413) (Table 4)(Fig. 2).

**Table 4:** Multiple Linear Regression with HQ-25 total score (as dependent variable)

Only sample of males									
	B	SE	β	t	p-value	95%IC Lower Limit	95%IC Upper Limit	Tolerance	VIF
MSBS total score	0.147	0.028	0.428	5.293	** < 0.001**	0.092	0.202	0.562	1.779
PHQ-9 total score	0.564	0.192	0.237	2.944	**0.004**	0.186	0.943	0.567	1.763
TEMPS-M Hyperthymic	− 0.266	0.133	− 0.123	− 2.003	**0.047**	− 0.529	− 0.004	0.971	1.030

Only sample of females									
	B	SE	β	t	p-value	95%IC lower limit	95%IC upper limit	Tolerance	VIF
MSBS Low Arousal	0.475	0.088	0.332	5.365	** < 0.001**	0.301	0.649	0.285	3.512
MSBS Disengagement	0.107	0.050	0.130	2.125	**0.034**	0.008	0.206	0.289	3.461
PHQ-9 total score	0.502	0.126	0.215	3.971	** < 0.001**	0.254	0.750	0.371	2.697
GAD-7 total score	− 0.354	0.138	− 0.140	− 2.559	**0.011**	− 0.625	− 0.082	0.363	2.755
TEMPS-M Irritable	0.206	0.081	0.094	2.543	**0.011**	0.047	0.365	0.801	1.248
TEMPS-M Anxious	0.168	0.077	0.087	2.166	**0.031**	0.016	0.320	0.675	1.482
TEMPS-M Depressive	0.176	0.084	0.098	2.102	**0.036**	0.012	0.340	0.498	2.010

*SE* standard error, *HQ-25* hikikomori questionnaire-25 items, *PHQ-9* patient health questionnaire-9, *MSBS* multidimensional state boredom scale, *TEMPS-M* munster temperament evaluation of memphis, Pisa, Paris and San Diego

In bold significant p-values

**Fig. 3:**
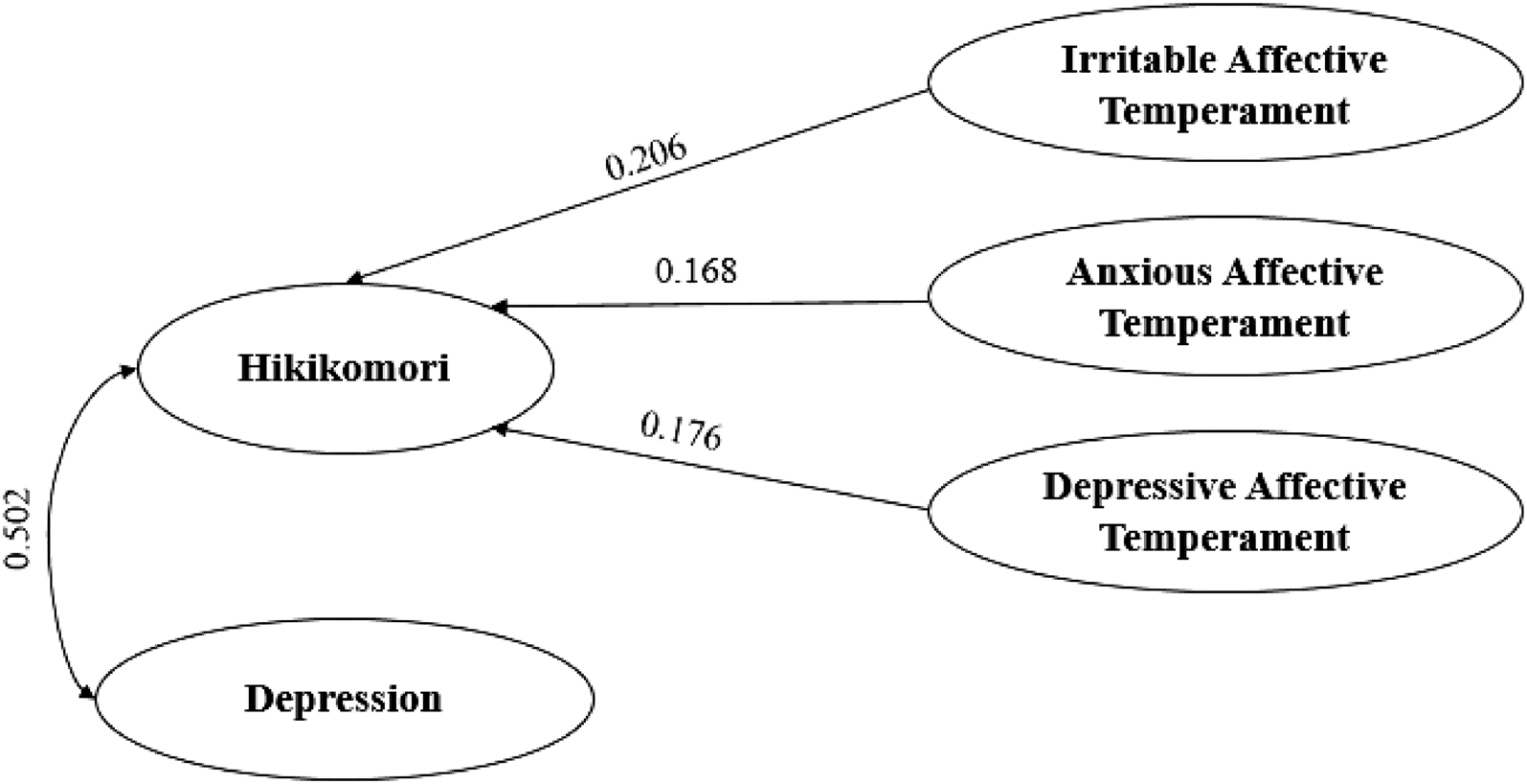
Graphical representation of relationships between Hikikomori, depression and affective temperaments (females)

Conversely, according to the multivariate regression model, among females, HQ-25 levels were positively predicted MSBS low arousal levels [B = 0.475; 95%CI = (0.301)–(0.649); p < 0.001], MSBS disengagement [B = 0.107; 95%CI = (0.008)–(0.206); p = 0.034], depressive levels, as measured by PH9 total score [B = 0.502; 95%CI = (0.254)–(0.750); p < 0.001]; and by irritable affective temperament [B = 0.206; 95%CI = (0.047)–(0.365); p = 0.011], anxious affective temperament [B = 0.168; 95%CI = (0.016)–(0.320); p = 0.031] and depressive affective temperament (B = 0.176; 95%CI = (0.012)–(0.340); p = 0.036]. While HQ-25 levels were negatively predicted by GAD-7 total score [B = -0.354; 95%CI = (− 0.626)–(− 0.082); p = 0.011]. These variables statistically significantly predicted Hikikomori-like social withdrawal symptomatology (F(7, 515) = 57.605, p < 0.001, R^2^ = 0.439) (Table 4)(Fig. 3).

When the multivariate linear regression model was run, by selecting only depressed individuals, HQ–25 levels were positively predicted by MSBS total score levels (B = 0.172; 95%CI = (0.098)–(0.245); p < 0.001], MSBS low arousal levels (B = 0.358; 95%CI = (0.114)–(0.602); p < 0.001], and by depressive affective temperament (B = 0.277; 95%CI = (0.094)–(0.459); p = 0.003]. While HQ-25 levels were negatively predicted by MSBS High Arousal levels [B = − 0.347; 95%CI = (− 0.620)–(− 0.074); p = 0.013]. These variables statistically significantly predicted Hikikomori-like social withdrawal symptomatology (F(4, 383) = 48.544, p < 0.001, R^2^ = 0.336) (Table 5).

**Table 5:** Multiple linear regression with HQ-25 total score (as dependent variable)—only sample of depressed individuals (PHQ ≥ 10)

	B	SE	β	t	p-value	95%IC lower limit	95%IC upper limit	Tolerance	VIF
MSBS Low Arousal	0.358	0.124	0.224	2.886	**0.004**	0.114	0.602	0.289	3.464
MSBS total score	0.172	0.038	0.457	4.565	** < 0.001**	0.098	0.245	0.173	5.796
MSBS High Arousal	− 0.347	0.139	− 0.188	− 2.500	**0.013**	− 0.620	− 0.074	0.305	3.279
TEMPS-M Depressive	0.277	0.093	0.138	2.983	**0.003**	0.094	0.459	0.813	1.230

*SE* standard error, *HQ-25* hikikomori questionnaire-25 items, *PHQ-9* patient health questionnaire-9, *MSBS* multidimensional state boredom scale, *TEMPS-M* munster temperament evaluation of Memphis, Pisa, Paris and San Diego

In bold significant p-values

A logistic regression analysis was performed to ascertain the effects of all types of five affective temperaments (as measured by TEMPS-M), on the likelihood of developing depression with Hikikomori-like social withdrawal symptomatology. The logistic regression model was statistically significant, χ2(1) = 4.423, *p* = 0.035. The model explained 12.3% (Nagelkerke *R*2) of the variance in depression with Hikikomori-like social withdrawal symptomatology and correctly classified 65.5% of cases. According to the logistic regression model, depression with Hikikomori-like social withdrawal symptomatology was significantly predicted by higher levels at TEMPS-M subscales which measures depressive and cyclothymic affective temperaments. Other affective temperaments did not show to be predictive of the onset of a depression with Hikikomori-like social withdrawal symptomatology (Table 6).

**Table 6:** Logistic Binary Regression within depressive sample with HK (presence/absence as dichotomous dependent variable)

	B	SE	Wald	Exp (B)	p-value	95%IC lower limit	95%IC upper limit
TEMPS-M Depressive	0.080	0.018	19.385	1.083	** < 0.001**	1.045	1.122
TEMPS-M Cyclothymic	0.035	0.017	4.407	1.033	**0.036**	1.002	1.069

*SE* standard error, *HQ-25* Hikikomori questionnaire-25 items, *TEMPS-M* munster temperament evaluation of memphis, Pisa, Paris and San Diego

In bold significant p-values

## Discussion

To the best of our knowledge, this is the first study investigating the relationship between affective temperaments and Hikikomori-like social withdrawal symptomatology in a cohort of young adults, by comparing clinically significant depressed versus not-depressed individuals and exploring differences between sexes. In particular, following more recent research directions which suggested to explore Hikikomori-like social withdrawal as a transdiagnostic specifier, particularly within the sample of individuals with depressive symptomatology [6, 34], our study specifically explored the association between Hikikomori as diagnostic specifier associated with depression and the identification of specific predominant associated affective temperaments. Overall, our findings found that, within the total recruited sample, HQ-25 mean total scores are overly higher compared to previous published studies carried out within an Italian sample [28, 35, 36], probably due to the younger age of our sample and the recruitment period which was after the COVID-19 pandemic. Comparable with previous studies [28, 35, 36], in our sample, males reported significantly higher HQ-25 scores, particularly in the subscales ‘isolation’ and ‘emotional support’, compared to females. In the total sample, Hikikomori-like social withdrawal symptomatology was found to be significantly predicted by higher levels of low arousal boredom (i.e., by manifesting dysphoria, feelings of emptiness and fatigue) and feelings of disengagement from meaningful and interesting life activities (as measured by MSBS). Moreover, Hikikomori-like social withdrawal has been significantly predicted by higher depressive levels, lower anxiety levels and by higher levels at irritable and depressive affective temperaments (as measured by TEMPS-M). Indeed, despite some studies having been carried out by exploring some child temperamental features and social isolation, there are no published studies specifically addressed to young adults [37, 38]. These studies found an association between child social isolation and the presence of the so-called behavioral inhibition temperament, i.e. the tendency to react following exposure to unfamiliar stimuli by developing anxiety and avoidance behavior [37, 38]. Akiskal already identified a possible association between social isolation and specific affective temperaments, such as cyclothymic (particularly in transient social isolation episodes), and depressive affective temperaments (more associated with the tendency to develop a social withdrawal) [39, 40]. Subjects with depressive affective temperaments tend to be sensitive to suffering, self-denying and devote themselves to others [40]. Their optimal balance is achieved when they are in harmony with others, adhering to social norms and roles [40]. According to this perspective, social isolation could be a maladaptive mechanism to escape suffering or the inability to find harmony with the world around them. In addition, such subjects are characterized by harm avoidance and low novelty seeking, which often leads the subject to a boring life and, hence, could result in a progressive isolation [40]. On the other hand, subjects with an irritable affective temperament, are characterized by the presence of irritable-lunatic mood with 'ill-humored joking' [30]. Such traits could result in disagreements with peers and social impairment leading to progressive social withdrawal both as distanced from others and as a maladaptive defense mechanism (such a person might feel not understood by others) [30]. This could be reinforced by the fact that such individuals have a tendency to brood and great impulsivity [30]. Disengagement in the process leading to boredom is characterized by a difficulty in the process of orienting and attributing attention to the environment resulting in a mismatch between fully experiencing an activity and paying attention to it [41, 42]. This process may explain why this dimension is related to social withdrawal. Indeed, the lack of attention to the environment results in a disinterest in what we find in the environment, with a progressive isolation. Finally, low arousal is connected with the proneness to boredom. In particular, subjects with a low arousal try to find some activities to enhance their arousal [43]. If this process is maladaptive, we can hypothesize that persistent low activation could lead to isolation through feelings of emptiness and fatigue, despite we should integrate these findings by using longitudinal cohort studies to demonstrate whether there is a causal relationship between boredom dimension and the onset of a HK-like social withdrawal within the depressed individuals.

Moreover, considering that our sample is mainly represented by females, we also carried out a sub-analysis assessing potentially sex-based differences in the clinical and predisposition to the development of a depression with or without a Hikikomori diagnostic specifier. In particular, according to our findings, within the male sample, Hikikomori-like social withdrawal conditions seemed to be significantly predicted by general higher boredom levels and the level of depressive severity. While only the presence of a predominant hyperthymic temperament seemed to be negatively associated with the presence of a Hikikomori-like social withdrawal, by suggesting a potential protective role, which should be further investigated and confirmed in larger longitudinal studies evaluating both depressed and not-depressed HK individuals and considering sex-based differences between both samples. Indeed, there are no published studies which allow us to confirm these findings, despite Akiskal previously suggested a possible association of transient social isolation episodes (not Hikikomori-like) and a predominant hyperthymic temperament, mainly occurring as a reaction to the social and seldom maladaptive consequences of their temperaments and the subsequent need to self-isolate in order to have not been exposed to a negative judgment from others [40]. However, social isolation among hyperthymic individuals, may indeed represent a transient reaction which rarely meets Hikikomori diagnostic criteria [6]. Furthermore, our findings reported that, among females, Hikikomori-like social withdrawal seemed to be significantly associated with low arousal and disengagement boredom levels, by depression severity and by irritable, anxious and depressive affective temperaments. Indeed, previous literature already documented that, among females, the most predominant affective temperaments are generally represented by anxious and depressive affective temperaments [20] by, hence, suggesting that probably the irritable affective temperament could indeed more likely be associated to the psychopathological trajectory leading to HK-like social withdrawal symptomatology [30]. However, also these preliminary findings should also be extensively confirmed and replicated in more larger sex-based cohort longitudinal studies, as there is no still published literature on the topic, either in depressed versus not depressed young adults.

Furthermore, after stratifying the entire sample according to the presence versus absence of a depressive symptomatology, according to our findings, depression associated with Hikikomori specifier seemed to be positively associated with higher general boredom levels, particularly low arousal boredom levels. Therefore, within the depressed sample, Hikikomori seemed to not be predicted by disengagement boredom levels as observed in the total sample. This could be explained by the fact that individuals in whom there is a greater component of the disengagement dimension, could display some difficulty in recognizing internal information (e.g., thoughts and emotions) [41]. On the other hand, this ability is often present in those suffering from depression, and this would result in the ability to direct attention to specific environmental elements, more likely responsible for the development of the depressive symptomatology. Moreover, the likelihood of developing Hikikomori-like social withdrawal symptomatology within the context of a depression seemed to be positively predicted by the presence of predominant depressive and cyclothymic affective temperaments, confirming data already observed by Akiskal [21, 30]. Generally, major depressive disorder (MDD) is associated with depressive and anxious temperament [20, 44]. Cyclothymic temperament is associated with the development of Bipolar Disorder (BD), in particular to type II [20, 44]. However, this temperament is also associated with forms of MDD lately evolving into BD, in those individuals who develop MDD but with a positive family history for BD and in atypical forms of MDD [20, 44]. Therefore, cyclothymic temperament could be associated with those clinical phenotypic depressive forms which could be different from the classic MDD clinical picture. Cyclothymic could evolve into depression associated with Hikikomori-like social withdrawal as a diagnostic specifier, manifested by mood swings, which could lead to increasingly frequent depressive episodes over time and, hence, resulting in the potential development of a progressive social and emotional isolation.

Therefore, based on our preliminary findings, one could argue that depression associated with Hikikomori-like social withdrawal symptomatology could represent a distinct type of depression which should be adequately investigated and clinically characterized from a diagnostic and therapeutic perspective, in order to build a personalized and tailored-based intervention. The association with specific affective temperamental profiles could also help clinicians in early identifying those depressed individuals at-risk to develop a clinical picture associated with Hikikomori specifier which, indeed, could potentially modify clinical course, outcomes and treatment strategies.

However, despite these preliminary and promising findings, our study has several limitations which should be adequately addressed and discussed before generalizing our results. Firstly, findings coming from the total sample could be influenced by sex unbalance, being our total sample mainly represented by females. Secondly, our sample could be influenced by the highly age-based selection, mainly represented by young adults (aged 18–35). Therefore, further studies should be carried out by including a more representative sample of individuals (independently by the presence of comorbid depressive symptomatology), in order to clearly confirm these findings only in primary Hikikomori and according to different age ranges. Conversely, when comparing both groups (depressed versus not-depressed), both samples are sex- and age-based homogeneously represented. Therefore, one could argue that findings coming from sub-analysis could be more easily generalizable to the sample of individuals affected by Hikikomori secondary to depression. Thirdly, the cross-sectional nature of our study does not allow us to draw up definitive conclusions regarding the relationship between depression and Hikikomori-like social withdrawal (i.e., identifying whether an individual is affected by a primary depression with Hikikomori or a depression secondary to Hikikomori). Fourthly, being our total sample mainly represented by females, our findings regarding the association between specific affective temperaments and Hikikomori-like social withdrawal symptomatology could be biased by the female-effect in temperamental profiles. Fifthly, our study is a nationwide population-based study and, hence, our findings could be influenced by selection bias, by the fact that our sample is a nonclinical one. Therefore, further larger longitudinal, multicentric and pan-European based studies should be conducted in order to replicate our preliminary findings as well as longitudinally identifying specific sex-based predictors influencing the clinical course, manifestation, treatment outcomes and prognosis of individuals affected by depression depending on the presence of Hikikomori diagnostic specifier. Furthermore, despite our preliminary findings also investigated as secondary exploratory outcomes, boredom dimensions and anxiety symptomatology, the cross-sectional nature of the study did not allow to draw uo definitive conclusions regarding the potential causal relationship between boredom dimension and subdimensions and the increased/decreased chance to develop Hikikomori-like social withdrawal both in depressed versus not depressed individuals, despite our findings could suggest also a potential association and role depending on the type of predominant affective temperament.

Overall, our preliminary findings could significantly help clinicians working with young adults manifesting depressive symptomatology by potentially shedding the light on the possible association between specific predominant affective temperamental profiles and the increased chance to develop a depression associated with the Hikikomori diagnostic specifier. However, our findings coming from a nationwide, Italy-based, nonclinical population-based study specifically recruiting young adults aged 18–35 which could indeed help in providing a current snapshot of the Italian situation regarding youth depression with/without Hikikomori, despite our findings should be extensively replicated in longitudinal clinical studies recruiting both primary and secondary Hikikomori subjects. An interesting result comes from our sex-based stratified sub-analysis, which suggested a potential different clinical phenotypization depending on the sex and also influenced by predominant affective temperament, which should also be confirmed and verified in larger longitudinal clinical studies. In particular, there is the need to confirm which affective temperaments could be protective (or risky) for the development of a Hikikomori-like social withdrawal symptomatology in depressed individuals. Early identification of affective temperament in patients with depression could help in predicting which will be the potential developing psychopathological trajectory leading to the onset of a Hikikomori-like social withdrawal associated with depressive symptomatology and which should be the tailored and personalized treatment to be adapted accordingly. Meanwhile, a comprehensive personological characterization of individuals who develop Hikikomori-like social withdrawal, considering both depressed versus not depressed individuals would be useful to better clinically characterize from a diagnostic and therapeutic perspectives these subjects, also investigating the (potential) mediatory role of the boredom dimensions as well as attachment style profiles and anxiety trait and state. Finally, following suggestions and research hypotheses of previous researchers [1, 2, 4, 10], it would be appropriate to clinically characterize depression associated with Hikikomori by identifying similarities and differences (if any) with the psychopathological construct of the Modern-Type Depression.

## Data Availability

The datasets used and/or analyzed during the current study are available from the corresponding author on reasonable request.
